# Optical Thickness-Encoded Suspension Array for High-Throughput Multiplexed Gene Detection

**DOI:** 10.3390/s19245425

**Published:** 2019-12-09

**Authors:** Huiying Ma, Xuejing Chen, Bangrong Lu, Yanhong Ji

**Affiliations:** 1 School of Physics and Telecommunication Engineering, South China Normal University, Guangzhou 510006, China; 2017021624@m.scnu.edu.cn (H.M.); 2016021660@m.scnu.edu.cn (B.L.); 2 Shenzhen Key Laboratory for Minimal Invasive Medical Technologies, Institute of Optical Imaging and Sensing, Graduate School at Shenzhen, Tsinghua University, Shenzhen 518055, China; chenxj15@mails.tsinghua.edu.cn

**Keywords:** suspension array, micro-quartz pieces, multiplexed DNA detection, optical thickness coding, dual wavelength digital holographic phase fluorescence microscope

## Abstract

We proposed a coding and decoding method of suspension array (SA) based on micro-quartz pieces (MQPs) with different optical thicknesses. The capture probes (cDNA) were grafted onto the surfaces of MQPs and specifically recognized and combined with the partial sequence of the target DNA (tDNA) to form a MQP-cDNA-tDNA complex. Quantum dot-labeled signal probes were then used to specifically recognize and bind another portion of the tDNA in the complex to form a double-probe sandwich structure. This optical thickness-encoded SA can be decoded and detected by a dual-wavelength digital holographic phase fluorescence microscope system. We conducted a series of DNA molecule detection experiments by using this encoding method. Control experiments confirmed the specificity of optical thickness-encoded SA in DNA detection. The concentration gradient experiments then demonstrated the response of the MQPs based SA to analyte concentration. Finally, we used the encoding method to detect three types of DNA in a single sample and confirmed the feasibility of the proposed optical thickness-encoded SA in multiplexed DNA detection. The detection results are stable, and the detection exhibits high specificity and good repeatability.

## 1. Introduction

As disease diagnosis and treatment progresses, the demand for the high-throughput and multiplex analysis of numerous biomolecules within a single sample has increased. Most of these diseases, including thalassemia, HPV infectious diseases, uveitis and HIV, are caused by gene deletion, gene translocation, gene mutation, or protein variation [1,2,3]. Many types of these diseases exist, but the quality of diagnosis of these diseases is limited by small sample volumes. In recent years, many multiplex detection techniques, such as multiplex quantitative polymerase chain reaction [4,5] and suspension array (SA) technologies have been used. SA technology is a multi-index analysis and detection platform, based on chip technology, flow cytometry, laser analysis, and high-speed digital signal processing [6,7]. The identification of different biomolecule binding events by using encoded microcarriers is the core of SA. The unique structural design of SA allows the simultaneous detection of multiple biomolecules in the same reaction system. Given that the detection process is conducted in a liquid-phase reaction system, SA exhibits higher sensitivity, faster reaction speed and shorter incubation time than a conventional solid-phase biochip due to the liquid phase reaction kinetics principle [8]. In addition, the preparation process of SA is simple, and the detection result is stable and easy to repeat. Therefore, this technology has been widely used in gene detection [8,9,10], protein analysis [11,12], hormone detection [13,14], cytokine detection [15], and immunological analysis [16,17].

Polystyrene microspheres are commonly used as microcarriers in a conventional SA technology. Microsphere-based multiplex detection relies on encoded microspheres. At present, several microsphere-coding methods have been developed, including fluorescence, Raman spectral and photonic crystal coding methods. The most common method of fluorescent coding is usually realized by doping organic dyes or quantum dots (QDs) into the microspheres. However, the stability of organic dyes coding methods is poor [18], and organic dyes are prone to fluorescence spectral overlap [19] and photobleaching. In addition, spectral interference could occur because both the coding and label signals are fluorescence spectra [20]. QDs coding method can also cause spectral crosstalk among QDs [21]. These drawbacks may cause decoding errors and low multiplexing capacity and sensitivity. Meanwhile, Raman spectral coding and photonic crystal coding methods have been extensively studied. However, the commonly used Raman molecules exhibit similar structures and the characteristic peaks of different Raman reporter molecules almost all concentrated at 500 cm^−1^–2000 cm^−1^. Considering that Raman molecules usually have several characteristic peaks, characteristic peaks could overlap when several Raman molecules encoded microspheres are used simultaneously in actual detection and subsequently reduce the encoding capacity [22,23]. The core of the photonic crystal coding method is to obtain a series of colloidal photonic crystal microspheres that exhibit different reflection spectra by changing the sizes of the nanoparticles that constitute the microspheres. The microspheres can then be encoded based on the peak position of the reflectance spectrum [24,25]. In the encoding process, the reflection spectrum peak must be located in advance, and this process is increasingly complicated in actual operation. Therefore, these coding methods have certain limitations in practical applications.

In our previous study, we proposed high-throughput detection coding and decoding techniques for SA [26,27], including laser-induced breakdown spectroscopy coding [28] and a dual-wavelength digital holographic phase and fluorescence microscope system (DW-DHPFM) for decoding optical thickness-encoded SA [29]. The DW-DHPFM system was used to image and decode the optical thickness of micro-quartz pieces (MQPs). Proteins and small molecules, such as herbicides, insecticides, and drugs, can be detected simultaneously by using this SA. 

In this work, an optical thickness-encoded SA for multiplexed detection of DNA molecules within a single sample with the DW-DHPFM system was proposed. The size of each MQP is 100 × 100 µm and the thickness is used as the coding element. For the encoding and decoding dimension, the thicknesses of the MQPs are relatively stable during the whole reaction, and thus greatly improving the decoding accuracy. In the experiment, we used the single-stranded DNA (ssDNA) of common clinical pathogens as target genes and designed corresponding capture and signal probes. In the coding process, different types of capture DNA were cross-linked onto MQPs with different optical thicknesses. Then, the target DNA was analyzed by decoding the optical thicknesses of the MQPs when the capture probe specifically bound to the target DNA. Given that another part of the target DNA sequence can specifically bind to the QD-labeled signal probes, the target DNA in the sample can be quantified by detecting the fluorescence intensity of the QDs. The axial resolution of the DW-DHPFM system established in our previous study is 0.41 µm, and the axial measurement range is about one synthetic wavelength 203.40 nm. Considering the distribution range of optical thickness standard difference (0.50~1.50 μm), the number of decoding barcodes is more than one hundred. Making it possible to detect large quantity of molecules at the same time. In addition, our DW-DHPFM system has two imaging paths, the phase imaging path and the fluorescence imaging path. The phase imaging path was used to decode the optical thickness-encoded microcarriers, and fluorescence imaging path was used to detect the quantitative information of the bound target DNA [29]. No mutual interference occurred between the two signals, and thus, decoding sensitivity and accuracy could be greatly improved.

## 2. Materials and Methods

### 2.1. Materials and Reagents

MQPs with different thicknesses of 52.30 ± 0.60, 71.50 ± 0.80, and 89.80 ± 0.80 µm were purchased from Shuyang Jingtong Quartz Technology Co., Ltd. (Suqian, China). Acetone, sulfuric acid (98%), and toluene were purchased from Confidence Hong Trading Co., Ltd. (Guangzhou, China). Hydrogen peroxide (30%), and sodium borohydride were purchased from Guangzhou Zixing Glass Instrument Co., Ltd. (Guangzhou, China). Borate standard buffer solution (pH = 9.18) was purchased from Xiamen Haibiao Technology Co., Ltd. Carboxyl water-soluble QDs were obtained from Wuhan Jiayuan Quantum Dot Technological Development Corporation (Wuhan, China), and 1-ethyl-(3-dimethyl aminopropyl) carbodiimide hydrochloride (98%) was purchased from Aladdin (Shanghai, China). γ—Glycidyl ether propyl trimethoxysilane (GPTMS) was purchased from Guangzhou Jianyang Biotechnology Co., Ltd. Target DNA, capture DNA, and signal probes used in the experiment were purchased from Invitrogen Trading (Shanghai) Co., Ltd. The specific base sequences are shown in Table 1.

### 2.2. Characterization

The morphology of MQP was investigated by a scanning electron microscope (Zeiss Gemini 500, Germany). The ultraviolet spectra of the QDs, signal probes (DNA), QD-labeled signal probes (QD-DNA), and QD-labeled signal probe filtrate after 3 ultrafiltrations were obtained by using a UV-visible spectrophotometer (TU-1810, Persee). The optical thickness-encoded SA was decoded and detected by a DW-DHPFM system consisting of two parts, namely, phase and fluorescence imaging paths. The optical thickness-encoded microcarriers were decoded by the phase imaging path, and the sequence information of the genes was determined by detecting MQP thickness. In the fluorescence imaging path, the quantitative information of the bound target DNA was detected by fluorescence intensity. 

### 2.3. Preparation of Quantum Dot-Labeled Signal Probes

**Figure 1:**
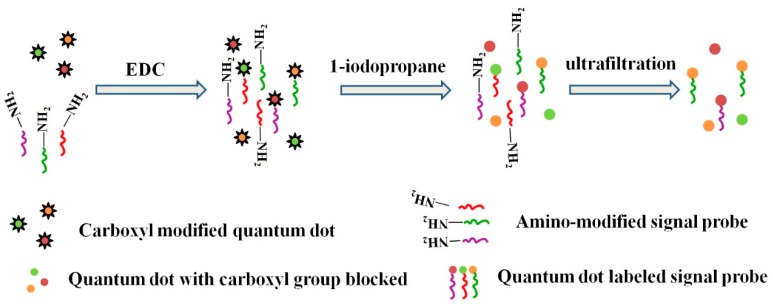
Schematic of quantum dot-labeling signal probe.

The 3′ amino-modified signal probes were labeled with carboxyl modified water-soluble QDs. The marking process is shown in Figure 1, and the main steps are shown as follows: (1) First, 8 µL of carboxyl-modified water-soluble QDs was added to a 500 µL centrifuge tube. Borate buffer was then added. The resulting 1 µM solution was stirred for 15 min. (2) Approximately 300 µL of 533 nM pDNA solution was added to the solution and stirred for 2 h. (3) Approximately 50 µL of 0.1 M EDC borate solution was added to the centrifuge tube and stirred for 8 h. (4) A QD-labeled signal probe (QD-pDNA) was obtained by filtering the solution through an ultrafiltration centrifuge tube (10 kd) and washing the wall of the centrifuge tube with PBS.

### 2.4. Encoding and Decoding of SAs

**Figure 2:**
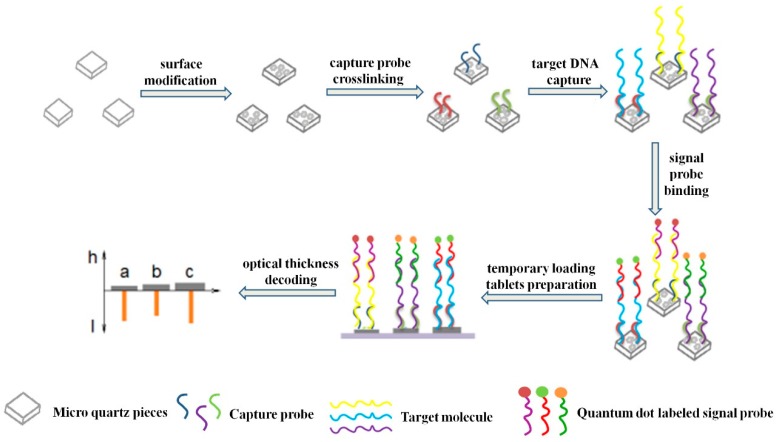
Schematic of the preparation and decoding of suspension arrays.

The overall encoding and decoding schemes are presented in Figure 2. First, the surfaces of MQPs with different thicknesses were modified with epoxy groups. Second, three types of capture probes were grafted on the surfaces of three types of MQPs with different thicknesses. Third, the target sequences were specifically recognized and bound by the capture probes. Fourth, QD-labeled signal probes were allowed to react with target DNA specifically recognized by the capture probes. Finally, the combined complexes (MQP-capture probe-target DNA-signal probe) were dropped on a glass slide and decoded by a self-build optical system.

The specific coding steps for each link are as follows: (1) MQP collection: MQPs attached to the plastics were placed in a 1.5 mL centrifuge tube, and 1 mL of acetone solution was added. The solution was ultrasonicated until the MQPs were completely scattered at the bottom of the centrifuge tube. (2) MQP cleaning: MQPs were successively washed with acetone, ethanol, and deionized water. (3) MQP hydroxylation: MQPs were dispersed into piranha lotion. After being soaked overnight, the upper piranha lotion was removed. And the MQPs were washed with ethanol and deionized water for the hydroxylation of the MQPs. (4) MQP epoxylation: epoxylated MQPs (MQPs-CH (O) CH-) were obtained by soaking the hydroxylated MQPs in 1% GPTMS toluene solution for 12 h at room temperature and washing them with ethanol and deionized water. (5) Capture probe grafting: 500 µL of 670 nM capture DNA was added to the centrifuge tube that contained MQPs-CH (O) CH- and shaken on a shaker for 8 h. The upper solution was then removed. The MQPs with the capture probes were soaked in 10% 1-iodopropane in ethanol for 1 h and successively washed with ethanol solution and deionized water. (6) Target DNA capturing: 500 µL of 670 nM target DNA solution was dispersed to the centrifuge tube containing MQPs-capture DNA and stirred for 8 h. MQPs-capture probe-target DNA complex was obtained by washing the solution with PBS (0.60M NaCl) solution. (7) Specific binding of the probe to the target DNA: The quantum dot-labeled signal probes were then transferred to the centrifuge tube containing the MQPs-capture probe-target DNA complex with a pipette and stirred for 8–12 h. The MQPs were then successively washed with PBS and deionized water. Finally, the MQPs were made into a temporary loading and placed on a self-build optical system for decoding.

### 2.5. Specificity and Gradient Detection

To explore the most appropriate concentration of signal probe DNA, we firstly conducted a concentration gradient experiment with the signal probe concentration as the variable. In this part, 525 nm QD-labeled HPV oligonucleotide sequence solutions with concentrations of 25.00, 12.50, 6.25, 3.09, and 1.54 nM and 771.25, 385.63, and 0 pM were tested as analytes. To verify the specificity of the optical thickness-encoded SA in DNA detection, we conducted a specificity experiment that included an experimental group and two control groups. In the experimental group, the HPV sequences were used as target DNA and the average optical thickness of MQPs is 52.30 ± 0.60 µm. In the control groups, we changed the HPV target sequences to the *Staphylococcus aureus* (gltS) and *Escherichia coli* 0157:H7 (stx2) target sequences. Then, to demonstrate the concentration response of SA, the concentration gradient experiment was designed. Eight groups of HPV target sequence solutions with different concentration, 25.00, 12.50, 6.25, 3.09, and 1.54 nM and 771.25, 385.63, and 0 pM, were tested as analytes.

### 2.6. Multiplexed Detection

We performed a series of experiments to demonstrate that the optical thickness-encoded SA can be used in multiplexed DNA detection. We used a single sample containing three types of DNA as analytes. Considering the practicality of the detection, we selected the DNA of three clinically common viruses and bacteria as target DNA, which are oligonucleotide sequences of HPV, *S. aureus* (gltS), and *E. coli* 0157:H7 (stx2). In the experiment, three types of capture DNA were grafted onto the surface of three types of MQPs with different average optical thicknesses (MQP1, 52.30 ± 0.60 µm; MQP2, 71.50 ± 0.80 µm; MQP3, 89.80 ± 0.80 µm). Namely, HPV capture probe-MQP1, *S. aureus* (gltS) capture probe-MQP2 and *E. coli* 0157: H7 (stx2) capture probe-MQP3; then, they were all placed into the single sample containing the three detected sequences. After the target DNA molecules were specifically recognized and bound by the capture probes, the supernatant solution was removed. The complex was then washed with PBS solution. Finally, the QD-labeled signal probes that specifically bound to the three target sequences were added (525 nm @ HPV, 565 nm @ *S. aureus* (gltS), and 645 nm @ *E. coli* 0157: H7 (stx2) signal probes).

### 2.7. Optical Decoding System

**Figure 3:**
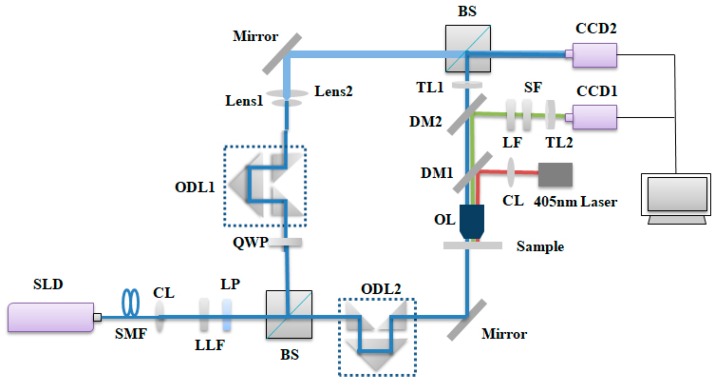
Schematic of the DW-DHM system SLD, superluminescent diode; SMF, single-mode fiber; CL, collimator; LLF, laser line filter; LP, linear polarizer; BS, beam splitter; QWP, quarter-wave plate; ODL, optical delay line; OL, objective lens; DM, dichroic mirror; LF, long-pass filter; SF, short-pass filter; TL, tube lens.

The decoding of optical thickness-encoded SA consisted of two parts: the thickness decoding of MQPs and fluorescence detection. The thickness decoding of the MQPs was achieved by a dual-wavelength digital holographic system (DW-DHM), and fluorescence intensity was detected with a fluorescence microscope (FM). The optical decoding system is shown in Figure 3. Two working wavelengths at 830 nm (λ1) and 833.40 nm (λ2) were obtained by band-pass filtering the beam from the superluminescent diodes (SLD) with two laser line filters (LLF, 830 and 852 nm). The center wavelength of the filtered spectrum was shifted to 833.40 nm by tilting the filter LLF2 with a center wavelength of 852 nm to a certain angle. A synthetic wavelength of 203.40 μm was then obtained for the axial height measurements. The DW-DHM system consisted of a Mach–Zehnder interferometer. Two sets of optical delay lines, namely, ODL1 and ODL2, were used to adjust the optical path length difference between the reference and sample arms. In the sample arm, the back focal planes of the objective lens (OL) coincided with the front focal plane of the lens barrel TL1 to counteract the spherical aberration introduced by the OL. In the reference arm, the reference light was adjusted by the mirror to incident CCD2. The reference light and the sample light interfere on the receiving surface of the CCD2, and the polarization components were adjusted to maximize the fringe contrast of holograms. Finally, holograms were captured by CCD2.

For the FM path, a 405 nm laser (ShanghaiXilong, 100 mW) was used as the excitation light source. The laser beam was reflected by the dichroic mirror (DM1) and uniformly incident to the sample. Excitation fluorescence was transmitted through DM1 and reflected by a dichroic mirror. The excitation fluorescence then passed through long and short pass filters. Finally, the tube lens was used to deliver fluorescence to CCD1, and the information of CCD1 and CCD2 was received and analyzed by the computer. 

## 3. Results and Discussion

### 3.1. Characterization of Encoded SA

**Figure 4:**
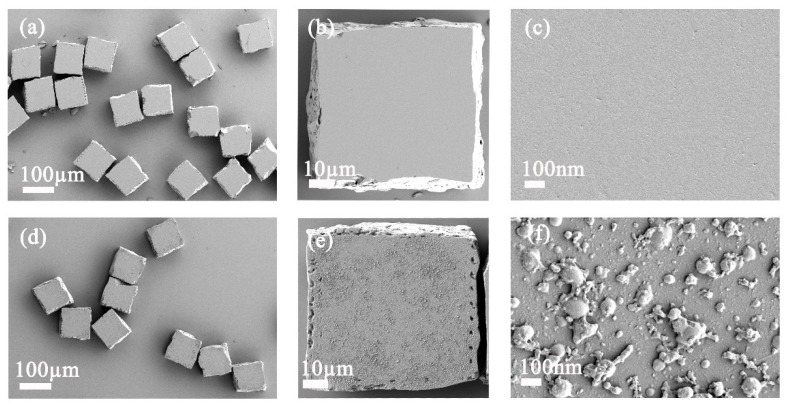
SEM images of initial MQPs and GPTMS modified MQPS. (**a**–**c**) SEM images of initial MQPs. (**d**–**f**) SEM images of GPTMS modified MQPs. The scale bars are 100 and 10 µm and 10 nm for (**a**,**d**); (**b**,**e**); and (**c**,**f**), respectively.

**Figure 5:**
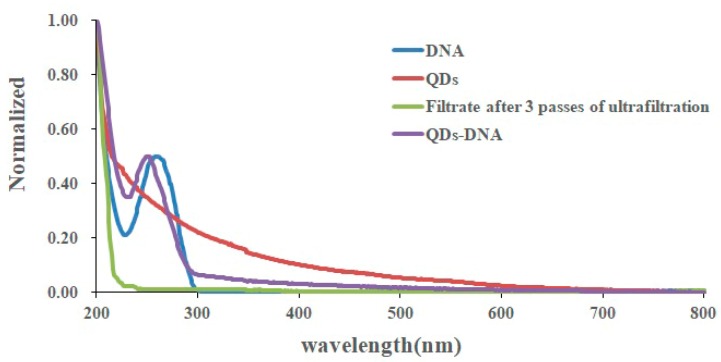
Normalized ultraviolet spectra of quantum dots (QDs), signal probes (DNA), quantum dot-labeled signal probes (QD-DNA), and quantum dot-labeled signal probe filtrate after 3 ultrafiltrations.

To illustrate the feasibility of the encoding method, we conducted a series of verification experiments. First, we selected three MQPs with different optical thicknesses, namely MQP1 (52.30 ± 0.60 µm), MQP2 (71.50 ± 0.80 µm), and MQP3 (89.8 ± 0.8 µm) as the SA carriers. The surfaces of the MQPs were modified with epoxy groups. GPTMS toluene solution was used, and three types of DNA capture probes were grafted on the MQPs. Figure 4 shows the SEM images of the MQPs. Figure 4a–c shows cleaned initial MQPs that were not modified with epoxy groups. Obviously, the unmodified MQPs show clean and smooth surface. Figure 4d–f shows the epoxy-modified MQPs, demonstrating the rough surfaces of the GPTMS-modified MQPs. Moreover, we can easily come to the conclusion that the modifying groups were uniformly distributed on the surfaces of the MQPs. We then labeled three types of signal probes with QDs of different emission wavelengths and measured the ultraviolet spectra of the QDs, signal probes, and QD-labeled signal probes with an ultraviolet spectrophotometer. To confirm that no free DNA was present in the QD-labeled signal probe solution, we ultrafiltered the QD-labeled signal probes three times with an ultrafiltration centrifuge tube and measured the ultraviolet spectrum of the filtrate. The normalized ultraviolet spectrum of QDs, signal probes (DNA), QDs-labeled signal probes, and QD-labeled signal probe filtrate after three iterations of ultrafiltration is shown in Figure 5. As shown in Figure 5, the ultraviolet spectra of DNA (blue) and QD-DNA (purple) exhibited peaks at 260 nm, and the wavelengths of the two peaks were very close. The ultraviolet spectra of QDs (red) and filtrate (green) showed no peaks at 260 nm. This result indicates that no free DNA existed in the filtrate and confirms that QDs were successfully labeled on the signal probes. In addition, the manufacturing process of the MQPs used in the experiment was relatively simple. These MQPs can be industrially cut by laser alone, are nontoxic, and easier to store than chemically synthesized nanoparticles or microspheres.

### 3.2. Specificity and Concentration Response of Optical Thickness-Encoded SA in DNA Detection

**Figure 6:**
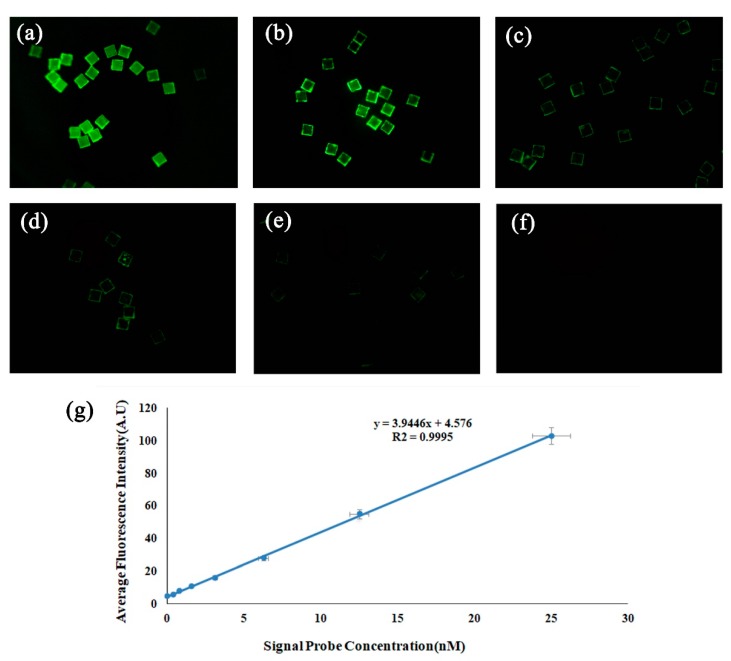
(**a**–**f**) Fluorescence images of reacted MQPs corresponding to signal probe labeled with 525 nM QDs at 25.00, 12.50, 6.25, 3.09, and 1.54 nM and 771.25, 385.63, and 0 pM. Size of field of view of (**a**), (**b**), and (**c**): 1.26 mm × 1.26 mm. (**g**) Concentration response curve obtained by fitting the analyte concentration to the mean fluorescence intensity.

In order to explore the most suitable concentration of signal probe DNA. A serial of gradient concentration experiments with the signal probe concentration as the variable were firstly conducted and the result is shown in Figure 6. According to the result and considering the cost of our research, in our other experiments, a signal probe concentration of 25 nM was used. 

**Figure 7:**
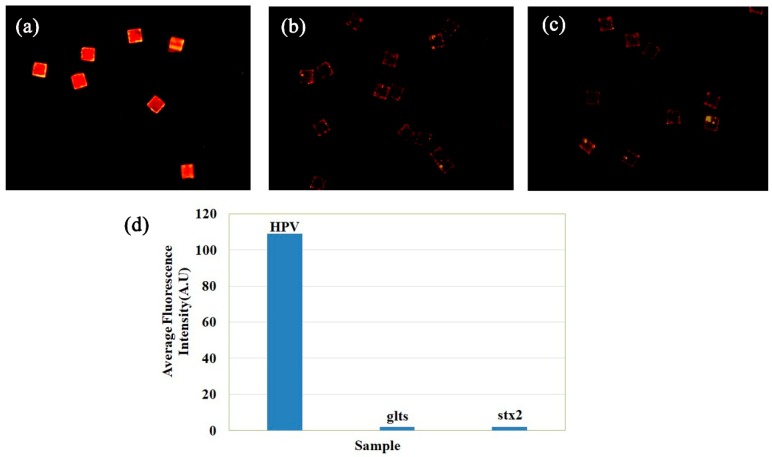
(**a**), (**b**), and (**c**) Fluorescence images of DNA using HPV probes to detect HPV, *Staphylococcus aureus* (gltS), and *Escherichia coli* 0157:H7 (stx2). Size of field of view of (**a**), (**b**), and (**c**): 1.26 mm × 1.26 mm. (**d**). The average fluorescence intensity of MQP reacted with HPV, *Staphylococcus aureus* (gltS), and *Escherichia coli* 0157:H7 (stx2).

In the specific detection process, we used a type of DNA capture and signal probe to detect three types of analytes. We used HPV probe and 605 nm QD-labeled HPV signal probes to detect and quantify HPV, *S. aureus* (gltS), and *E. coli* 0157:H7 (stx2) DNA, respectively. The test results are shown in Figure 7. The analytes in Figure 7a–c are HPV, *S. aureus* (gltS), and *E. coli* 0157:H7 (stx2) signal probes, respectively. As shown in Figure 7, the MQPs of Figure 7a exhibited relatively strong fluorescence brightness, and the MQPs were evenly dyed. The edge of the MQPs in Figure 7b,c was bright. However, almost no fluorescence was observed in the middle area. Analysis reasons are as follows: (1) The capture probes and signal probes of HPV cannot recognize *S. aureus* (gltS) and *E. coli* 0157:H7 (stx2) DNA. (2) The edge portions of the MQPs in Figure 7b,c are bright because the physical cutting generates rough edges, and the QDs were easily adsorbed on the rough surface by nonspecific adsorption. However, the physical nonspecific adsorption of the rough edge of MQPs does not affect the quantitative analysis ability of the method because we only calculated the middle area (each side of the MQP is removed by 5 μm) of MQPs during the quantitative target analyte information detection. Figure 7d demonstrated the quantified fluorescence intensity of signal probes after reacting with target HPV DNA, *S. aureus* (gltS) DNA, and *E. coli* 0157:H7 (stx2) DNA, respectively. The experimental results showed that a DNA capture probe and signal probe can be used only to hybridize the specific DNA with high efficiency and that our optical thickness-encoded SA exhibited good specificity in DNA detection.

**Figure 8:**
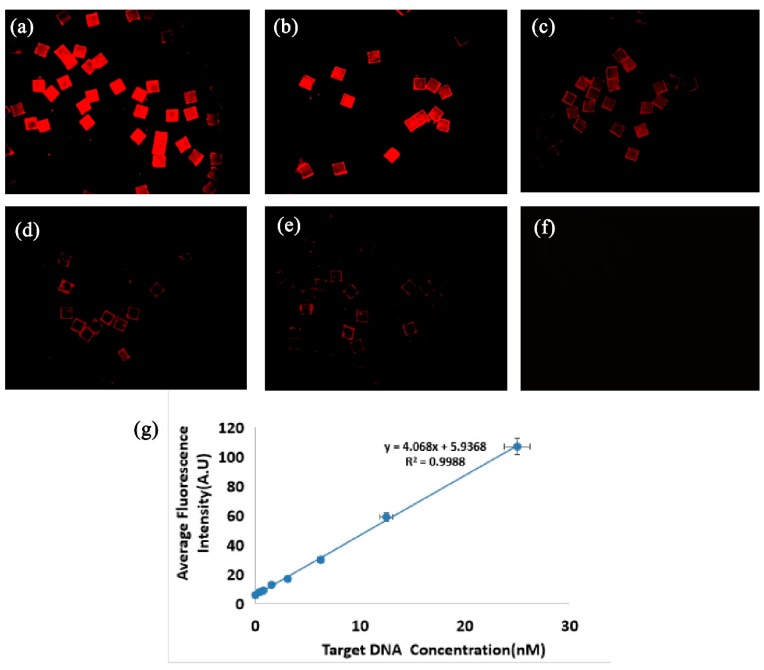
(**a**–**f**) Fluorescence images of reacted MQPs corresponding to target HPV DNA at 25.00, 12.50, 6.25, 3.09, and 1.54 nM and 771.25, 385.63, and 0 pM. Size of field of view of (**a**), (**b**), and (**c**): 1.26 mm × 1.26 mm. (**g**) Concentration response curve obtained by fitting the analyte concentration to the mean fluorescence intensity.

For the gradient detection response experiments, target HPV sequence at 25.00, 12.50, 6.25, 3.09, and 1.54 nM and 771.25, 385.63, and 0 pM were tested as analytes. The test was aimed at demonstrating the concentration response of the MQP-based SA. Figure 8 shows the fluorescent image of analyte-captured MQPs measured by the DW-DHPFM system at each concentration. Figure 8 shows that the fluorescence intensity gradually decreased with analyte concentration. Figure 8g is the response curve showing the relationship between the average fluorescence intensity and analyte concentrations. The average fluorescence intensity indicates the average intensity of each pixel, excluding the edge, for each MQP. The standard difference fluorescence intensities of each concentration were calculated from 100 different MQPs. The standard difference of average signals acquired from five replicate experiments was also calculated. As shown in Figure 8g, the vertical error bar represents the intra-sample standard difference, and the horizontal error bar represents the inter-sample standard difference. The standard deviation obtained by repeated measurements was 0.13 (A.U) to blank samples. According to the principle of triple standard difference method, the detection limit of 4.52 × 10^−11^ M was calculated.

### 3.3. Application of Optical Thickness-Encoded SA in Multiplexed DNA Detection

**Figure 9:**
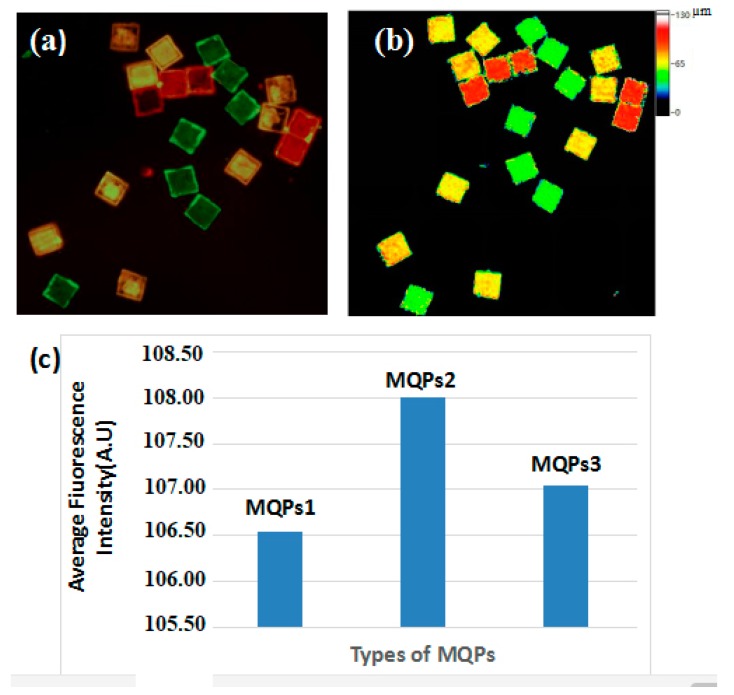
(**a**) Fluorescence images and (**b**) reconstructed optical thickness image of the reacted MQP1, MQP2, MQP3 in multiplexed analysis. Size of field of view: 1.26 mm × 1.26 mm. (**c**) The average fluorescence intensity of three types of MQPs.

To confirm the practical application of optical thickness-encoded SA in multiplexed DNA detection, we grafted three types of DNA capture probes on three different MQPs (MQP1, 52.30 ± 0.60 µm; MQP2, 71.50 ± 0.80 µm; and MQP3, 89.80 ± 0.80 µm). Specifically, the capture probes of HPV-DNA, *S. aureus* (gltS) DNA, and *E. coli* 0157:H7 (stx2) DNA were grafted onto MQPs1, MQPs2, and MQPs3, respectively. The three target DNA molecules were then mixed together as analytes. The MQPs coupled with the capture probes were added to the analyte to specifically bind to the target DNA. The signal probes of HPV, *S. aureus* (gltS), and *E. coli* 0157:H7 (stx2) DNA were then labeled with 525, 585, and 625 nm QDs, respectively, and the signal probes were mixed after being labeled. Finally, three types of MQPs that bound with the target DNA were added to the mixture with three QD-labeled signal probes to specifically bind to the signal probes. Figure 9 shows the fluorescence image and optical thickness decoding results for the three specific binding reactions. The colors of the three fluorescence in Figure 9a were produced by the QDs with three different emission wavelengths (525, 585, and 625 nm). Figure 9b shows the reconstructed optical thickness image. In the figure, one color represents a type of MQP with the same optical thickness. The MQPs shown in green, yellow, and red fluorescence in Figure 9a correspond to (green) MQPs, (yellow) MQPs, and (red) MQPs in Figure 9b, and their optical thicknesses were 50, 70, and 90 µm, respectively. The three MQPs were detected by the decoding system. The MQPs of the same color in Figure 9a had the same optical thickness. Only one fluorescence was found on the same MQP after the exclusion of edge pixels. This result confirms that few nonspecific reactions occurred on the middle areas of the MQPs. As shown in Figure 9c, the fluorescence intensities of the MQPs were measured by the FM path of the DW-DHM decoding system, and the average fluorescence intensity determines the quantitative information of the analytes. Therefore, this experiment demonstrates the feasibility of our optical thickness-encoded SA for DNA multiplexed detection.

## 4. Conclusions

We proposed a novel multiplexed DNA detection method. Compared with traditional DNA detection methods, the proposed encoding scheme of the optical thickness-encoded SA exhibits many advantages. In this encoding approach, MQPs with different optical thicknesses are used as microcarriers; the optical thickness of MQPs is used as the encoding signal, and the fluorescence on the signal probe is used as the label signal. Therefore, no interference will be found between the encoding and label signals. Given that the thicknesses of the MQPs were stable in the whole reaction process, decoding accuracy and sensitivity can be greatly improved. Furthermore, through specific experiments and concentration gradient experiments, we demonstrated the practicability of the encoding method in multiplexed DNA detection. Some potential challenges may exist in the further practical application of this method. For example, the encoding capacity is limited. However, these problems can be solved by combining other encoding dimensions with optical thickness-encoding dimensions or constructing a stable and accurate optical thickness measurement system. Therefore, the optical thickness-based SA encoding method has broad application prospects in multiplexed biomolecule detection.

## Figures and Tables

**Table 1:** Oligonucleotide DNA strand base sequence.

Virus/Pathogenic Bacteria	Sequence Name	Base Sequence (5’*—*3’)
HPV	Target sequence 1	AGATATTTGGAATAACATGACCTGGATGCA
	Capture probe 1	TTATTCCAAATATCT(A_10_)-NH_2_
	Signal probe 1	NH_2_-(A_10_)TGCATCCAGGTCATG
Staphylococcus aureus (gltS)	Target sequence 2	ACGTTGCATCGGAAACATTGTGTTCTG
	Capture probe 2	TTCCGATGCAACGT)-NH_2_
	Signal probe 2	NH_2_-CAGAACACAATGT
Escherichia coli 0157: H_7_ (stx2)	Target sequence 3	TACTCCGGAAGCACATTGCTGAATC
	Capture probe 3	TGCTTCCGGAGTA-NH_2_
	Signal probe 3	NH_2_-GATTCAGCAATG
